# The differential expression of microRNA-143,145 in endometriosis

**Published:** 2014-08

**Authors:** Bingbing Zheng, Xiangyang Xue, Yiping Zhao, Jing Chen, Chao-Yi Xu, Ping Duan

**Affiliations:** 1 *Department of Obstetrics, First Affiliated Hospital of Wenzhou Medical University, Zhejiang, China.*; 2 *Department of Microbiology, Wenzhou Medical University, Zhejiang, China.*; 3 *Department of Gynecology, Zhuji People's Hospital, Zhejiang, China.*; 4 *Department of Reproductive Medicine, Second Affiliated Hospital of Wenzhou Medical University, Zhejiang, China.*; 5 *Department of Gynecology, Second Affiliated Hospital of Wenzhou Medical University, Zhejiang, China.*

**Keywords:** *MiRNA* (*microRNA*), *MiR*-*143*, *MiR*-*145*, *Endometriosis*

## Abstract

**Background:** Recent studies showed that inappropriate expression of microRNAs (miRNAs) is strongly associated with tumor-related processes in humans (2-9,11-17).

**Objective:** To understand the changes of miRNAs in endometriosis.

**Materials and Methods: **With real-time RT-PCR, we investigated the miR-143 and miR-145 expression in eutopic (EU, n=2) and ectopic endometrium (EC, n=11) (from women with endometriosis) (as well as EU+EC, n=11), along with the normal endometrium (EN, n=22) (from women without endometriosis, but with leiomyoma).

**Results: **We did not find that the expression of miR-143 and/or miR-145 in EN or EC changed with menstrual cycle. But our results showed the miR-143 was up-regulated in EC (p=0.000) compared to EN. The miR-143 was also up-regulated in EU, but the difference did not reach statistically significance (p=0.053). Compared to EU, the expression of miR-143 in EC was up-regulated (p=0.016). MiR-145 had the similar characteristic to miR-143. The miR-145 was up-regulated in both EU (p=0.004) and EC (p=0.000) in compared to EN group. When compared with EU, the miR-145 in EC was up-regulated (p=0.008).

**Conclusion:** In conclusion, the miR-143 and miR-145 may play a certain role in the development and progression of endometriosis.

## Introduction

Endometriosis, defined as the growth of endometrial tissue outside the uterine cavity, is a common gynecological disease. The prevalence of pelvic endometriosis approaches 6-10% in the general female population; in women with pain, infertility, or both, the frequency is 35-50% (1). Nevertheless, the etiology and pathogenesis of endometriosis have remained uncertain. The retrograde displacement of eutopic endometrium into the pelvis and its subsequent implantation on peritoneal surfaces is a leading theory as to the etiology of this condition. 

Extensive investigations have been performed to characterize the differences between the eutopic and ectopic endometrium, including the gene expression changes. MicroRNAs (miRNAs) are endogenously expressed short noncoding RNAs, 18–25 nucleotides in length, which suppress protein translation through binding to target messenger RNAs (mRNAs).They are expressed at specific stages of tissue development or cell differentiation, and have large-scale effects on the expression of a variety of genes at the post-transcriptional level. Recent studies suggest that miRNAs contribute to the development of various cancers. Many miRNAs are highly tissue specific and play a significant role in oncogenes, cancer metastasis and tumor invasion (2). It has also been found that most miRNAs target both oncogenes and tumor suppressor genes, so they have dual nature (3). 

Recently, it has been found that miRNA overexpression could result in down-regulation of tumor suppressor genes, whereas miRNA under expression could lead to oncogene up-regulation (-). For example, miR-143, which is down-regulated in colon cancer, suppresses ERK5 (5). MiR-143 and miRNA-145 are located within approximately 1.8 kb of each other in the chromosome 5q32 region, which led us to speculate that both precursors originate from the same primary miRNA (6). According to the published on EMs, we find that miR-143 expression in different types of cancer spectrum is inconsistent. Recently, Akao *et al* found that miR-143 and -145 were down-regulated in colon cancer (5, 6). 

Further, they examined the expression levels of the both miRNAs in three human cancer cell lines, SW480, DLD-1, andCOLO201, and even various kinds of human cancer cell lines. Expectedly, all the tested showed a considerable low-expression of both miRNAs, whereas the expression in the human normal tissue was fairly strong. Then they used the miR-143 or -145 precursor miRNAs for transfection of the human colon cancer DLD-1 and SW480 cells, the cell growth of both cell lines was significantly inhibited in a dose-dependent manner at 48 h. Also, both MiR-143 and miR-145 are frequently down regulated in tumors deriving from breast, lung, colon, the gastrointestinal system, ovary, cervix, bladder tissue and in human cancer cell lines, suggesting anti-oncogenic nature of miR-143 and -145 (7, 8). However, Ohlsson *et al* and Filigheddu *et al* both found that miR-143 and -145 are up-regulated in ectopic endometrium compared with paired eutopic endometrium in women with endometriosis (9, 10).

Zhang *et al* demonstrated that the level of miR-143 is dramatically increased in metastatic HBV-HCC of both p21-HBx transgenic mice and HCC patients (11). Moreover, they found that local liver metastasis and distant lung metastasis are significantly inhibited by blocking miR-143 in p21-HBx transgenic mice, showing that overexpression of this miRNA favors liver tumor cell invasive and metastatic behavior. In addition, Bloomston *et al* and Szafranska *et al* both found that miR-143 and -145 are up-regulated in pancreatic ductal adenocarcinoma (12, 13). Even though endometriosis is a benign disease, it has the similar behavior to metastatic HBV-HCC, including local invasion and distant metastasis. 

In this study, we employed stem-loop RT-PCR to quantify the miR-143 and miR-145 expression in endometrium of women with endometriosis, along with the endometrium of women without endometriosis. Our aim was to investigate the miR-143 and miR-145 expression in endometriosis and their relationships to clinicopathological variables in the patients.

## Materials and methods

This is a prospective study. The patients were diagnosed with endometriosis based on disease history, laboratory examination of CA125, radiology and clinical examinations.

**Patients and Samples**

The tissues were collected at the University of Wenzhou Medical College affiliated Second Hospital with written informed consent from patients. The exclusion criteria were the patients whose uterine leiomyomas were submucosal in location, or complicated with endometrial polyp, endometrial hyperplasia, endometritis, adenomyosis were excluded. Patients who were not normo-ovulatory with regular menstrual cycles or had received hormone therapy were also excluded. 

Portions of endometrium from women without endometriosis, but only with leiomyoma (EN) (total 22 cases; n1 (proliferative phase) =10; n2 (secretory phase) =11; n3 (proliferative -secretory phase) =1), paired eutopic and ectopic endometrium (EU and EC) (11 cases, including n1=7; n2=3; n3=1), ectopic endometrium (EC) (11 cases, tissues without the paired eutopic endometrium; n1=3; n2=8), eutopic endometrium (EU) (2 cases, tissues without the paired ectopic endometrium; n2=2) were collected from premenopausal women who were scheduled to undergo hysterectomy for indications related to symptomatic leiomyomas or endometriosis, respectively. 

All of the uterine leiomyomas were intramural in location. Endometriomas were removed by excision of the entire cyst wall, preserving normal ovarian tissue. No patients were receiving hormone therapy at the time of the study or in the previous three months. The patients’ age ranged from 25-51 years, with an average of 42 years. On the basis of their last menstrual period and endometrial histology, the tissues were from three kinds of phases of the menstrual cycle. All samples were histologically verified. Immediately after collection, the tissues were snapped frozen and kept in liquid nitrogen until RNA extraction could be performed, or used for cell isolation and culturing. Samples were homogenized by spertel under sterile conditions before total RNA isolation. 

Total RNA was isolated from the cells by using Trizol containing phenol/ guanidium isothiocyanate with DNase I treatment. RNA concentration and purity were assessed by UV spectrophotometry (A260: A280 >2.0; A260: A230 >1.8). RNA integrity was checked by using electrophoresis, and only no degraded RNA with any DNA contamination signs was processed.

**Reverse transcriptase reactions**

We designed miR-143,miR-145 and U6 stem-loop RT primers (Table I) according to the method developed by Chen *et al* and purchased the primers from Invitrogen (Invitrogen Biotechnology Co., Ltd, Shanghai, China) (14). To generate cDNA of miR-143, miR-145 and U6, 1μg of RNA and 10 μl DEPC H_2_O were first denatured at 70^o^C for 10 min before quenching on ice, and then 50nM stem-loop RT primer with 1mM final of each of the four deoxynucleotide triphosphates, 2 U/*μ*l ribonuclease inhibitor, 5 U/*μ*l M-MLV reverse transcriptase and 1*×*M-MLV RT buffer (Toyobo Co., Ltd, Japan) were added together to make up a final volume of 20 *μ*l reaction mix. 

The reaction mix was incubated in MJ Mini Gradient Thermal Cycler (Bio-Rad Laboratories, Inc., California, USA) for 30 min at 16^o^C, 30 min at 42^o^C. The reverse transcriptase was inactivated at 75^o^C for 15 min and then held at -20^o^C. A 20μl reaction system was used to perform real-time quantitative PCR.

**Real-time PCR analysis for miRNAs expression**

The miR-143 and miR-145 and endogenous control U6 primers (Table I) were purchased from the Invitrogen. The real-time PCR was performed using relative quantification protocol in a Roche Light Cycler 480(Roche, Basel, Switzerland). The 20 μl PCR included 1µl of RT product, 10µl 1× SYBR Green I Mastermix, 1.2 µM specific forward primer, 1.2 µM common reverse primer (Toyobo Co., Ltd, Japan). 

The reactions were incubated in a 96 well plate at 95^o^C for 2 min, followed by 40 cycles of 95^o^C for 15 s, 60^o^C for 1 min, and 72^o^C for 30 s. All reactions were run in triplicate. The results of the expression were obtained as a relative copy number by using the Ct value of the measured miRNA. The expression of each miRNA relative to U6 was determined using the 2^-ΔCT^ method (15). The following equations were applied to calculate: R_1_= the expression of the target gene in a sample. R_2_= the relative expression of the target gene in a sample versus a control in comparison to a reference gene (only applied in paired samples).

△CT=CT_miRNA_ - CT_U6_

△△CT= (CT_miRNA_-CT_U6_) ectopic -(CT_miRNA_-CT_U6_) eutopic

R_1_= 2^-^^△^^CT                    ^(1)

R_2_=2^-^^△△^^C                    ^ (2)

R_2_= R_1(EC)_ / R_1(EU)_

**Statistical analysis**

Data from real-time RT-PCR experiments were expressed as the P50 (P25, P75), given the non-normalized distribution. Student’s t-test was performed using the SPSS 16.0 statistical software package (SPSS, Inc., Chicago, Illinois, USA). Nonparametric test was used for statistical evaluation of miRNA expression. Wilcoxon rank sum test was used for all the comparison pairs, and Wilcoxon signed-rank test was only used for the comparison between paired EU and EC tissues. Tests were two-sided and differences with significance were accepted at p≤0.05. 

## Results

Using equation, we got the expression of miR-143 and/or miR-145 in all the samples (1). After comparison, in EN group, the expression levels of miR-143 and/or miR-145 in proliferative endometrium were up-regulated compared to secretory endometrium, but the difference did not reach statistically significance (p=0.085, 0.173, respectively Table II). Conversely, in EC group, proliferative endometrium was down-regulated compared to secretory endometrium, the difference also did not reach statistically significance (p=0.173, 0.557, respectively Table II). 

**Comparison among EN, EU and EC group (Using Wilcoxon rank sum test)**

Compared to EN, the expression level of miR-143 in EU was up-regulated, but the difference did not reach statistically significance (p=0.053). And the expression of miR-143 in EC was up-regulated (p=0.000). Compared to EU, the expression of miR-143 in EC was up-regulated (p=0.005). MiR-145 had the similar expression characteristic. Compared to EN, the expression level of miR-145 in EU was up-regulated (p=0.004). And also the expression of miR-145 in EC was up-regulated (p=0.000). Compared to EU, the expression of miR-145 in EC was up-regulated, but the difference did not reach statistically significance (p=0.020). The data are shown in table III.

**Comparison between paired EU and EC tissues (Using Wilcoxon signed-rank test)**

As described in the materials and methods section, R_2_ was presented only in paired EU and EC tissues. The value of R_2_* >*1.0 was considered to represent increased expression of miR-143 and/or miR-145 in EC tissues to the paired EU tissues, and R_2_* <*1.0 represent decreased expression. 

To further compare the overall level of miR-143 and/or miR-145 expression in EC tissues to the paired EU tissues, we got a second analysis using R_2_. The results showed that the miR-143 was up-regulated in EC compared to EU (p=0.016), and also the miR-145 was up-regulated (p=0.008). The data are shown in Table IV.

**Table I:** Specific stem-loop RT and PCR primers for miR-143, miR-145 and U6

**Primer**		**Sequence**
miR-143		
	RT stem-loop primer	5'-GTCGTATCCAGTGCAGGGTCCGAGGTATTCGCACTGGATACGACGAGCTACA-3'
	Forward primer	5'- ATGGTTCGTGGGTGAGATGAAGCACTG-3'
	Reverse primer miR-145	5′-GTGTCGTGGAGTCGGCAATTC-3′
	RT stem-loop primer	5'-GTCGTATCCAGTGCAGGGTCCGAGGTATTCGCACTGGATACGACAGGGATTC-3′
	Forward primer	5'- ATGGTTCGTGGGGTCCAGTTTTCCCAG-3′
	Reverse primer	5′-GTGTCGTGGAGTCGGCAATTC-3′
U6		
	RT stem-loop primer	5'- CGCTTCACGAATTTGCGTGTCAT -3'
	Forward primer	5'- GCTTCGGCAGCACATATACTAAAAT -3'
	Reverse primer	5'- CGCTTCACGAATTTGCGTGTCAT -3'

**Table II:** Comparison between proliferative and secretory endometrium respectively in EN and EC group [P50 (P25, P75)]

		**N**	**miR-143**	**p-value**	**miR-145**	**p-value**
EN						
	Proliferative phase	10	0.0305 (0.0216- 0.0578)		0.0038 (0.0034-0.0098)	
	Secretory phase	11	0.0205 (0.0182- 0.0292)	0.32	0.0032 (0.0013-0.0083)	0.09
EC						
	Proliferative phase	10	0.1138 (0.0757-0.1720)		0.0150 (0.0117-0.0276)	
	Secretory phase	11	0.1439 (0.0955-0.2014)	0.12	0.0212 (0.0175-0.0276)	0.24

Statistics: Wilcoxon rank sum test

**Table III:** Comparison among EN, EU and EC group [P50 (P25, P75)]

**Group**	**N**	**miR-143**	**miR-145**
EC	22	0.1283 (0.0868-0.1977)	0.0199 (0.0131-0.0280)
EU	13	0.0354 (0.0337-0.0542)	0.0095 (0.0058-0.0165)
EN	22	0.0271 (0.0202- 0.0440)	0.0038 (0.0024-0.0075)

Statistics: Wilcoxon rank sum test and Wilcoxon signed-rank test (for EU and EC)

**Table IV:** Comparison between paired EU and EC tissues [P50 (P25, P75)]

**Group**	**N**	**miR-143**	**miR-145**
EC	11	0.1283 (0.0893-0.1940)	0.0256 (0.0162-0.0430)
EU	11	0.0454 (0.0353-0.1349)	0.0095 (0.0059-0.0268)
R2	---	3.6243 (1.2838-4.4361)	3.1983 (2.7840-3.4023)

Statistics: Wilcoxon signed-rank test

## Discussion

In the preliminary study to this work, we performed miRNA microarray technology to identify the miRNAs differentially expressed in EN and paired EU/EC endometrium. And we have selected miR-143 and miR-145 here for further analyses (data not shown). In previous studies, miR-143 and miR-145 were also found differentially expressed in endometriosis, although some with a bit difference from our results (9, 10, 16-18).

In our study, we analyzed three types of samples, even paired EU and EC samples to identify the differential expression of miR-143 and miR-145.MiR-143 was up-regulated both in EU and EC samples compared to EN, although in EU the difference did not reach statistically significance. We firstly took a small sample into account. Moreover, miR-143 was up-regulated in EC compared to EU. Obviously, the expression of miR-143 in EN, EU and EC took on an uptrend. We drew a similar conclusion with miR-145.

As shown above, we found that miR-145 in EC group was up-regulated compared to EU group, but the difference did not reach statistically significance (p=0.020) when we used the Wilcoxon rank sum test. Furthermore, we got a new result when using a second statistical method - Wilcoxon signed-rank test, and the difference reached statistically significance (p=0.008). MiR-145 came to a same conclusion when using the two methods. Finally, we adopted the second statistical method because it used paired EU/EC endometrium from the same patients, thus avoiding the variables attributable to heterogeneous genetic background between individuals and the effects of estrogenic stimulation during different menstrual phases. Also the result is in line with the previous two studies (9, 10).

Burney *et al* reported significant dysregulation in gene expression signatures during the proliferative, early secretory (ES) and mid-secretory phases of the menstrual cycle in the endometrium from women with versus without endometriosis (19). They found that the ES phase involved the greatest number of statistically significant and differentially expressed genes of the three phases. Furthermore, they demonstrated a miRNA expression profile only in ES phase. But they had not reported dysregulation in miRNA expression signatures during the three different phases. In the present study, we analyzed proliferative versus secretory endometrium respectively in EN and EC group. The results showed the miR-143 and/or miR-145 in proliferative endometrium was up-regulated compared to secretory endometrium in EN group, but proliferative endometrium was down-regulated compared to secretory endometrium in EC group. All the differences did not reach statistically significance. 

The results had not yet found that the expression of miR-143 and/or miR-145 in EN or EC changed with menstrual cycle. The reason of the result may be attributed to the small sample. And maybe the result represents the truth. We also saw a pair of opposite results, so we suspected ectopic endometrium may play a more important role in the development and progression of endometriosis.

Recently, several studies showed gene expression differences between EU and EC samples (-). The differences reported in these studies include genes encoding proteins. They participate in the processes of cell adhesion, extracellular matrix remodeling, migration, proliferation and so on. These mechanisms hypothesized to be responsible for the establishment of ectopic endometrial implants. So the lack of knowledge about the targets for miR-143 and miR-145 hampered a full understanding on the biological functions and clinicopathological features. 

Referring to the studies which had been reported, we found that fibronectin type III domain containing 3B (FNDC3B), which regulates cell motility, was identified as the direct and functional target of miR-143 (11). And up-regulation of miR-143 expression transcribed by nuclear factor kappa B (NF-кB) promotes cancer cell invasion and metastasis by repression of FNDC3B expression. Another study also reported that FNDC3B was down-regulated in tumor cells with high metastatic potential. We suspected FNDC3B may also be the direct and functional target of miR-143 in endometriosis which had the similar clinicopathological features to malignant tumors, such as local invasion and distant metastasis.

Taken together, the results imply that the miR-143 and/or miR-145 may play a certain role in the development and progression of endometriosis. The body of expression data for miR-143 and miR-145 in endometriosis suggests that they may have a diagnostic and therapeutic potential. However, in the future, more and more studies should be performed to discover the relationship between miR-143/miR-145 and clinicopathological features of endometriosis, such as disease stages or clinical parameters, especially CA125 in endometriosis.
